# The Evolving Role of Calcium Channel Blockers in Hypertension Management: Pharmacological and Clinical Considerations

**DOI:** 10.3390/cimb46070377

**Published:** 2024-06-22

**Authors:** Kamryn E. Jones, Shaun L. Hayden, Hannah R. Meyer, Jillian L. Sandoz, William H. Arata, Kylie Dufrene, Corrado Ballaera, Yair Lopez Torres, Patricia Griffin, Adam M. Kaye, Sahar Shekoohi, Alan D. Kaye

**Affiliations:** 1 School of Medicine, Louisiana State University Health Sciences Center, Shreveport, LA 71103, USAclh005@lsuhs.edu (S.L.H.);; 2 School of Medicine, St. George’s University, True Blue, West Indies FZ818, Grenada; 3 Department of Anesthesiology, Louisiana State University Health Sciences Center, Shreveport, LA 71103, USA; corrado.ballaera@lsuhs.edu (C.B.);; 4 Department of Pharmacy Practice, Thomas J. Long School of Pharmacy and Health Sciences, University of the Pacific, Stockton, CA 95211, USA; akaye@pacific.edu; 5 Departments of Anesthesiology and Pharmacology, Toxicology, and Neurosciences, Louisiana State University Health Sciences Center, Shreveport, LA 71103, USA

**Keywords:** calcium channel blocker, hypertension, angiotensin-converting enzyme inhibitors, angiotensin receptor blockers

## Abstract

Worldwide, hypertension is the leading risk factor for cardiovascular disease and death. An estimated 122 million people, per the American Heart Association in 2023, have been diagnosed with this common condition. It is generally agreed that the primary goal in the treatment of hypertension is to reduce overall blood pressure to below 140/90 mmHg, with a more optimal goal of 130/80 mmHg. Common medications for treating hypertension include calcium channel blockers (CCBs), angiotensin-converting enzyme inhibitors, angiotensin receptor blockers, beta-blockers, and diuretics. CCBs are one of the most widely studied agents and are generally recommended as first-line therapy alone and in combination therapies. This is largely based on the vast knowledge of CCB mechanisms and their minimal side effect profile. CCBs can be separated into two classes: dihydropyridine and non-dihydropyridine. Non-dihydropyridine CCBs act on voltage-dependent L-type calcium channels of cardiac and smooth muscle to decrease muscle contractility. Dihydropyridine CCBs act by vasodilating the peripheral vasculature. For many patients with only mild increases in systolic and diastolic blood pressure (e.g., stage 1 hypertension), the medical literature indicates that CCB monotherapy can be sufficient to control hypertension. In this regard, CCB monotherapy in those with stage 1 hypertension reduced renal and cardiovascular complications compared to other drug classes. Combination therapy with CCBs and angiotensin receptor blockers or angiotensin-converting enzyme inhibitors has been shown to be an effective dual therapy based on recent meta-analyses. This article is a review of calcium channel blockers and their use in treating hypertension with some updated and recent information on studies that have re-examined their use. As for new information, we tried to include some information from recent studies on hypertensive treatment involving calcium channel blockers.

## 1. Introduction

Hypertension is typically defined as sustained systolic blood pressure of above or equal to 130 mmHg and/or sustained diastolic blood pressure above or equal to 80 mmHg [1,2,3]. Around one billion adults have hypertension worldwide, with this prevalence consistent among all socioeconomic and income statuses [3]. It is the leading comorbidity, second to smoking, in causing death and poor cardiovascular health, as well as being a common factor in contributing to the development of stroke, myocardial infarction, and heart and renal failure [3]. Although there are multiple etiologies of hypertension, non-modifiable risks for developing high blood pressure include gender, age, race, and heredity. Modifiable risk factors include smoking, lack of exercise, excessive salt intake, obesity, and increased alcohol consumption [4]. Essential hypertension is high blood pressure with no apparent secondary cause, while secondary hypertension is directly related to an underlying medical condition [3]. Hypertension that remains significantly above normal, with the use of three maximum doses of antihypertensive drugs of different classes, is defined as resistant hypertension [3]. Those with resistant hypertension display significant levels of end-organ damage or remodeling, including left ventricular hypertrophy, carotid plaques and intima-media thickening, and retinal and kidney involvement [5].

The pathophysiology of developing hypertension involves the alteration of many homeostatic mechanisms. The renin–angiotensin–aldosterone system (RAAS), as well as the sympathetic nervous system, plays significant a role in their pathophysiologic responses in sustaining high blood pressure [6]. Sympathetic stimulation, through the neurotransmitter noradrenaline, contributes to hypertension mainly through its actions on the heart and kidneys [6]. The kidneys maintain blood pressure through the regulation of the reabsorption and excretion of sodium and water. Changes in cardiac output and peripheral vascular resistance are also major contributors to regulating arterial pressure. As the kidneys sense an increase in intravascular volume and sodium concentration, the RAAS system is suppressed, causing an increase in diuresis and natriuresis [6]. Yet, as the body rids itself of excess sodium and water, the RAAS system is also activated as the juxtaglomerular cells sense a decrease in sodium and volume entering the kidneys, which also leads to an increase in sympathetic tone (via renal baroreceptors) [6]. As this happens, renin cleaves angiotensinogen into angiotensin I, leading to high levels of angiotensin II which results in various effects. Angiotensin II leads to peripheral vasoconstriction, increased sodium reabsorption, and the release of antidiuretic hormones that also increase water reabsorption. The RAAS and sympathetic nervous systems are constitutively activated and inactivated to maintain homeostasis regarding volume and sodium concentration, but sustained high blood pressure can shift the homeostatic threshold higher, leading to chronic high blood pressure. Increased salt intake also plays a major role in hypertension. Usually, cardiac receptor reflexes inhibit renal sympathetic activity, leading to the elimination of excess sodium. There are a handful of medications that are used solely or in combination to lower or inhibit the systems in the body that sustain high blood pressure. Targets include the renin–angiotensin system (RAAS) and the sympathetic activation of smooth muscle which increases vascular tone. These medications also seek to inhibit the remodeling of the heart, which is a major contributor to cardiovascular disease that can lead to complications such as myocardial infarction and aortic dissection. Treatment also includes considering a person’s atherosclerotic cardiovascular disease (ASCVD) risk, which aids in tailoring treatment to an individual’s personal risk factors [7]. For example, many studies have shown that the Black population benefits more from CCBs and diuretics. Comorbidity of kidney disease and heart failure can lead to the use of ACE inhibitors or ARBs as a first line, while diuretics can be used to aid other drugs in volume control by promoting natriuresis and diuresis [7]. This, combined with lifestyle modifications, yields the greatest sustained benefit in lowering blood pressure. First-line medications include beta-blockers, angiotensin-converting enzyme inhibitors, angiotensin II receptor blockers, loop and thiazide diuretics, and dihydropyridine CCBs. CCBs are one of the most intensely studied antihypertensive drugs, with plenty of evidence backing their benefits in lowering blood pressure, and they are generally recommended as first-line therapy or included in therapies with other drugs [7]. Dihydropyridine CCBs vasodilate blood vessels by binding to vascular smooth muscle L-type calcium channels [7]. They can be combined with most of the other first-line antihypertensive drugs, which explains their common use. Non-dihydropyridine CCBs function on cardiac calcium channels, reducing heart rate and contractility [8]. In this review, therefore, we discuss the pharmacological effects of CCBs and their evolving role in the treatment of hypertension (Table 1). The two classes of calcium channel blockers, dihydropyridines (DHPs) and non-dihydropyridines (Non-DHPs), are summarized in Table 1.

## 2. Methods

This is a narrative review. The sources for this review are as follows: searching on PubMed, Google Scholar, Medline, and ScienceDirect and using the keywords: calcium channel blocker; hypertension; angiotensin-converting enzyme inhibitors; angiotensin receptor blockers.

### 2.1. Mechanism of Action

**Figure 1:**
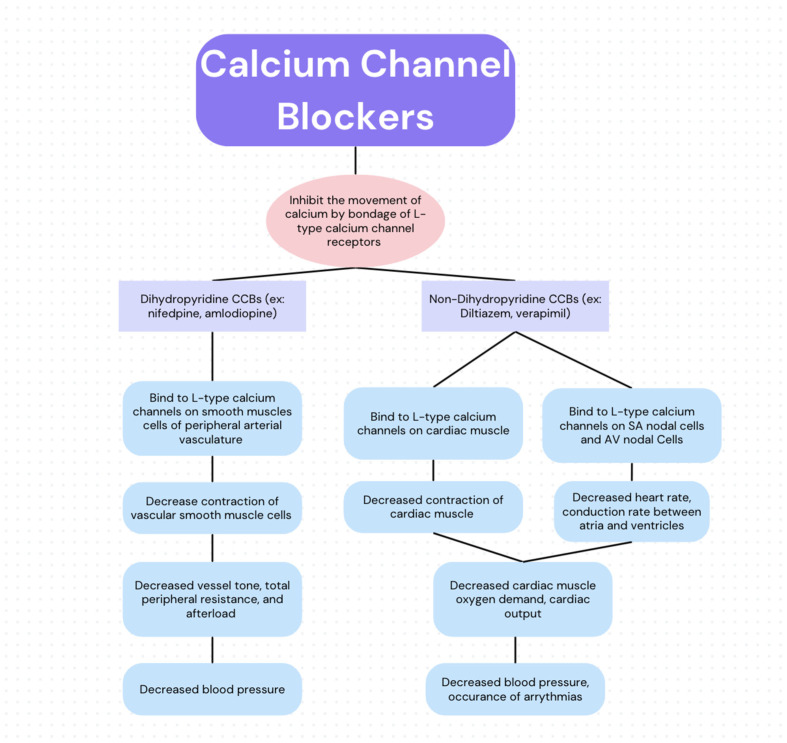
Schematic of CCBs’ mechanism of action.

CCBs function by inhibiting the movement of calcium by binding to L-type voltage-gated calcium channel receptors [9,10]. While all CCBs share this mechanism of action (Figure 1) and therefore have some vasodilatory effects, they can be separated into two overarching categories: dihydropyridines (DHPs) and non-dihydropyridines (non-DHPs). These categories are distinguished based on their primary physiological function, which is determined by their preferential binding to receptors in cardiac muscle or vascular smooth muscle [11,12]. DHPs, such as nifedipine and amlodipine, act primarily as vasodilators of the peripheral vasculature. This is accomplished via preferential binding to and blocking of L-type calcium channels located in the tunica media of the peripheral vasculature [13]. The subsequent decrease in the influx of calcium prevents the contraction of smooth muscle cells, thereby causing vasodilation. This causes decreased vessel tone, total peripheral resistance, and afterload, which therefore lowers blood pressure, hence their use in treating hypertension, migraines, and post-intracranial hemorrhage-associated vasospasms [14]. Alternatively, non-DHPs (e.g., verapamil and diltiazem) primarily function via preferential inhibition of L-type calcium channels of the myocardial intrinsic conduction system (e.g., the sinoatrial node and atrioventricular node) and the inhibition of cardiac myocytes [10,15]. Sinoatrial node inhibition causes a decrease in heart rate. When coupled with an overall decrease in cardiac myocyte contractility, there is a negative chronotropic and ionotropic effect that decreases cardiac muscle oxygen demand. Atrial ventricular node inhibition decreases the conduction rate between the atria and ventricles, which is particularly useful in managing supraventricular arrhythmia. This process aids in the treatment of hypertension, decreases heart strain by reducing oxygen demand, and aids in rate control in tachyarrhythmias.

### 2.2. Pharmacodynamics

CCBs are administered orally, or intravenously, and metabolized in the liver through hepatic first-pass metabolism by cytochrome P450 3A4 (CYP3A4) [14]. Analysis of the oral distribution of CCBs found the highest bioavailability (BA) in nifedipine (52.6%) while that of nicardipine and diltiazem was low (4.8% and 11.9%, respectively), suggesting high first-pass extraction in the liver or small intestine before reaching the systemic circulation [16]. In contrast, clevidipine is metabolized by plasma esterases. CCBs are then distributed via protein binding and are highly lipophilic, contributing to their large volume of distribution. CCBs are then excreted renally following hepatic metabolism [9]. Other formations of CCBs have demonstrated increased fecal excretion compared to renal excretion, although this is an uncommon means of elimination of most CCBs [17].

The biochemical structure of CCBs has been adjusted in order to allow for increased absorption by the body. In the process of synthesizing DHPM (dihydropyridine Ca channel blockers), scientists created a molecule with evidence of antihypertensive abilities; however, these molecules lacked in vivo antihypertensive activity. By adjusting various molecular groups on the original scaffolding structure of nifedipine, scientists were able to achieve better activity of these medications when administered orally [18,19].

CCBs allosterically bind to specific subunits of the Ca channel in order to establish their downstream effects of preventing Ca entry into cells [20]. Removal of the sequence resulted in the inability of verapamil and diltiazem to bind [20]. Similarly, dihydropyridines (DHP) bind allosterically to the same subunit of the Ca channel as non-dihydropyridines (NDHP). One study demonstrated this by visualizing amlodipine and diltiazem binding to distinct sites on the same subunit, Ca_V_Ab [20]. CCBs may act as competitive inhibitors against other subclasses of CCB. Kraus (1998) demonstrated that diltiazem partially blocks the binding site of other Ca channel-blocking drugs such as phenylalkylamines (PAA), another class of voltage-gated CCB, supporting that it is a competitive inhibitor with PAAs for this site [20,21].

### 2.3. Administration

CCBs are offered in both intravenous and oral formulations. The CCBs that have intravenous formulations include diltiazem, nicardipine, verapamil, and clevidipine [22].

When multi-drug therapy is indicated, Ca channel blockers may be administered with dual single-pill combination therapy. For hypertensive patients with cardiovascular comorbidities, reviews recommend dual single-pill combination therapy with a renin–angiotensin system (RAS) blocker, angiotensin-converting enzyme (ACE) inhibitor, or angiotensin receptor blocker (ARB) with a calcium channel blocker (CCB) or thiazide [23]. If target blood pressure is not achieved with a dual-drug combination, administration of a RAS blocker, CCB, and thiazide/thiazide-like diuretic in a single pill is recommended [24]. Similarly, this review also concluded that single-pill combinations resulted in greater reductions in blood pressure in patients with uncomplicated hypertension [23].

The timing of administration also plays a role in the efficacy of these types of medication. Studies have shown that CCBs are more effective when taken at bedtime rather than in the morning. Nighttime dosing results in a reduced risk of peripheral edema [25].

### 2.4. Duration of Action

CCBs can be further categorized by their duration of action including short-acting (nifedipine, diltiazem, and verapamil), long-acting modified release (nifedipine gastrointestinal therapeutic system, nifedipine CCB, and sustained-released verapamil), and inherently long-acting (amlodipine) [26]. Short-acting CCBs may have a time-to-peak effect ranging from 0.5 h to 1.5 h. Long-acting CCBs may have a time-to-peak effect ranging from 1 h to, in amlodipine’s case, up to 6–12 h. Short-acting nifedipine has a half-life of 2–4 h, while verapamil and diltiazem have half-lives of 6–8 h [27]. Long-acting modified-release CCBs, such as nicardipine, nifedipine SR, amlodipine, sustained-release verapamil, and sustained-release diltiazem, have a half-life of 12–24 h, with that of amlodipine being around 35–50 h [27]. Therefore, many of the long-acting modified release CCBs can be taken once a day, while the short-acting forms are taken up to three times a day.

CCBs can also be grouped into generations based on their characteristics. The first generation comprises fast-acting drugs like nifedipine and nicardipine, which have a quick onset but a brief duration of vasodilation [27]. Second-generation CCBs such as ER nifedipine, felodipine, and benidipine are released more slowly but still have a short activity span. Moving to the third and fourth generations, these CCBs display traits such as heightened vascular selectivity, reduced sympathoexcitation, and increased lipophilicity [27]. These characteristics result in a slower onset of action but a prolonged duration of vasodilation. They also tend to cause fewer side effects like peripheral edema and have a wider range of applications, including managing conditions like heart failure [27]. Specifically, third-generation CCBs include amlodipine and azelnidipine, while the fourth generation encompasses lacidipine, cilnidipine, and lercanidipine [27].

### 2.5. Dosing

CCBs, along with thiazide diuretics, ACE inhibitors, and ARBs, are recommended initial monotherapy treatments for essential hypertension, although questions arise when considering how high to titrate CCBs before adding a second agent [26,28]. While dose titration does yield better results, it also comes with a greater number of side effects which often serve as a trigger for drug discontinuation, reduction in dose, or the addition of a second drug class [24]. To limit this risk, CCBs are commonly used in combination with other BP-lowering drug classes. Dual drug treatment via the addition of an alpha antagonist, a beta-blocker, a diuretic, and/or an ACE inhibitor (or an ARB) to a CCB regimen can further reduce BP beyond the rates of monotherapy [24].

The dosing of CCBs for the treatment of hypertension varies depending on the specific drug and patient characteristics. The recommended starting dose for amlodipine is typically 5 mg once daily, for uncomplicated hypertension, which can be titrated up to 10 mg once daily based on individual patient response [29]. For other CCBs like nifedipine, the extended-release formulations are often initiated at 30 mg once daily, with a possible dose escalation to 60–90 mg once daily as needed [27]. It is important to note that dosing may need to be adjusted in certain populations such as elderly patients or those with renal impairment to ensure optimal efficacy and safety. Additionally, combination therapy with other antihypertensive agents may be considered for patients with resistant hypertension or multiple comorbidities, following current guidelines and individualized patient management plans. These dosing recommendations are supported by evidence-based guidelines and recent clinical studies in the field of hypertension management.

### 2.6. Side Effects and Contraindications

All subclasses of CCBs exhibit some degree of vasodilatory effect on peripheral vessels and therefore can exhibit similar side effects if their serum concentration exceeds therapeutic dosing ranges, although DHPs are more commonly associated with vasodilatory side effects [30]. The vasodilatory effects of CCBs can precipitate headaches, flushing, and hypotension, although peripheral edema is the most common side effect that impacts continued usage of these drugs [31,32]. As disproportionate changes in arteriolar resistance occur, there is an increase in precapillary hydrostatic pressure, which promotes fluid shifting into the interstitial compartment [30]. Women are more likely to experience CCB-related edema, and other associations include age, CCB dosage, and drug choice [24]. Nifedipine has the strongest vasodilatory effect and therefore has the closest association with peripheral edema. Studies show that the prevalence of peripheral edema is reduced when a DHP-CCB is given in combination with an ACE inhibitor or ARB, which vasodilates and increases postcapillary hydrostatic pressure, bringing the transcapillary pressure to equilibrium [33,34].

Non-DHPs’ negative chronotropic and ionotropic effects can precipitate bradycardia and worsen cardiac output. Therefore, their use is contraindicated in patients who are taking beta-blockers as it increases the risk of sinus bradycardia, atrioventricular blocks, and QT prolongation [35]. Negative ionotropic effects of this drug class also contraindicate their use in heart failure patients, particularly those with left-sided ventricular dysfunction [36,37,38]. Non-DHPs are also associated with constipation in up to 25 percent of patients [32]. Other associated side effects with all classes of CCBs include gastroesophageal reflux due to relaxation of the esophageal sphincter smooth muscle and gingival hyperplasia (Table 2).

### 2.7. Drug Interactions

CCBs’ interaction with other drug classes can alter the serum concentration of both the CCB and/or the co-administered drug. As a substrate of cytochrome P450 3A4, CCBs’ plasma levels can be altered if administered with inducers or inhibitors of CYP3A4. Grapefruit juice, a widely recognized CYP3A4 inhibitor, can interact with calcium channel blockers, potentially elevating the concentration of the medication in the bloodstream. This elevation may lead to perilous side effects like hypotension [39]. Additionally, inhibitors of CYP3A4, including certain CCBs, can heighten the blood levels of statins due to drug interactions. Consequently, there is a heightened risk of adverse events such as acute kidney injury when CYP3A4-metabolized statins are co-prescribed with CCBs that inhibit CYP3A4 [40].

Metabolism of immunosuppressants, such as cyclosporin and tacrolimus, can be altered related to coadministration with CCBs. While the administration of beta-blockers and DHPs yields additive hypotensive effects, beta-blockers combined with non-DHPs such as verapamil or diltiazem lead to negative inotropic and chronotropic effects that may cause significant AV nodal blockade, through which heart block, bradycardia, and cardiac conduction abnormalities can manifest [32].

## 3. Calcium Channel Blockers and Hypertension

CCBs are used in the treatment of hypertension as they induce vasodilatory actions, which lower total peripheral resistance, thereby lowering blood pressure [20]. In this regard, the decrease in blood pressure as a result of CCBs is more prominent in hypertensive patients than in normotensive patients, indicating that CCBs can be considered useful anti-hypertensive agents [41,42]. The efficacy of this drug class could be related in part to varying degrees of vessel affinity for CCBs, with one study finding that the vessels of hypertensive rats have a greater affinity for CCBs than those of normotensive rats [43]. Longstanding hypertension can lead to serious cardiovascular events and, ultimately, heart failure [44]. Updated in January 2022, an ongoing study, by the Department of Neurology at Sichuan University, compares randomized controlled trials comparing CCBS with other antihypertensive classes as first-line agents in reducing the incidence of major adverse cardiovascular events. They included cases with at least 100 randomized hypertensive participants and a follow-up of at least two years [45]. Their review found that reducing blood pressure using CCBs is as effective as doing so via other anti-hypertensive agents in the prevention of new-onset heart failure [45]. Moreover, CCBs have been shown to significantly decrease the risk of stroke and cardiovascular mortality as compared to the use of beta-blockers or ARBs [45,46,47,48,49]. One study even found that chronic treatment with CCBs in post-myocardial infarction (MI) patients reduced left ventricle (LV) dilation, improved LV function, prevented cardiac fibrosis and hypertrophy, and reduced heart rate [50]. Of note, only long-acting CCBs have been found to reduce left ventricular hypertrophy; their short-acting counterparts do not show the same effect [51]. These effects result in decreased myocardial oxygen demand [41,52]. Thus, CCBs are associated with cardioprotective properties against cardiac remodeling subsequent to pathological processes induced by hypertension.

Various studies have been conducted to evaluate significant differences between CCB monotherapy versus combination therapy with other antihypertensive agents. Overall, combination therapies are more effective in reducing blood pressure than monotherapies. Generally, for effective CCB combination therapy, the drug that is often chosen for coadministration acts on the renin–angiotensin–aldosterone system (RAAS), particularly angiotensin-converting enzyme (ACE) inhibitors and angiotensin receptor blockers (ARBs) [39]. Dihydropyridine CCBs induce RAAS, leading to edema as a potential side effect, although it is noted that the addition of an ACE inhibitor or ARB to CCB therapy can provide post-capillary vasodilation to reduce edema by decreasing intracapillary pressure [33,53]. Moreover, CCBs may strengthen the anti-hypertensive effects of ACE inhibitors by promoting a negative sodium balance and increased angiotensin-II levels [53].

The use of two CCBs, one dihydropyridine (DHP) and one non-dihydropyridine (NDHP), was evaluated as a potential combination therapy in the treatment of hypertension [54]. An analysis comprising randomized clinical trials found that dual CCB therapy with a DHP and an NDHP significantly decreases systolic and diastolic blood pressure when compared to monotherapy with either subset. The study further identified a potentially safer adverse effect profile with this combination including a lack of electrolyte abnormalities normally associated with diuretic use. This type of combination therapy might be considered in populations where agents affecting RAAS are contraindicated, such as patients with chronic kidney disease, although more studies are warranted to understand and evaluate this theory more thoroughly [54]. While dual CCB therapy has been shown to decrease blood pressure, the American Heart Association has stated that it is “premature from a purely blood pressure perspective to recommend the use of same-class combinations over the use of agents from different classes” in the treatment of resistant hypertension [55]. Therefore, CCBs are typically used in combination with anti-hypertensive drugs of another class, such as ACE inhibitors or ARBs, when it comes to treating refractory hypertension.

A bit of controversy was introduced during the late 1990s and early 2000s with studies examining the effects of long-acting versus short-acting calcium channel blockers [56]. One study revealed that the utilization of short-acting calcium antagonists correlated with a heightened risk of experiencing a cardiovascular event [22]. These results underscore the necessity of performing additional clinical trials on short-acting calcium channel blockers to evaluate the comparative cardiovascular effects of different antihypertensive agents.

One recurrent question regarding hypertension treatment is the effect of race or ethnicity on the type of antihypertensive medication that should be selected by providers. One study focused on a subset of patients in the COACH (Combination of Olmesartan Medoxomil and Amlodipine Besylate in Controlling High Blood Pressure) study [57]. The subset of patients included those with diabetes, Blacks, elderly (≥65 years) patients, and those who are overweight/obese with a BMI ≥ 30 kg m^−2^. They found that CCB monotherapy produced a higher percentage of target blood pressure achievements in the Black patient population over combination therapy with an ARB [34]. However, this study also found that, ultimately, combination therapies achieved a higher proportion of target blood pressure compared to monotherapy, regardless of race. Another study found that the Black population quantitatively has a greater response to CCB monotherapy in terms of lowered systolic and diastolic blood pressures, but qualitatively, there is not a significant difference in blood pressure response between the Black and White populations [58]. Therefore, these studies highlight the fact that clinical decisions on the type of anti-hypertensive therapy used should not be based on race or ethnicity but rather on the overall clinical picture of the patient.

## 4. Discussion

Worldwide, hypertension is the leading risk factor for cardiovascular disease and death, and an estimated 122 million people, per the American Heart Association in 2023, have been diagnosed with this common condition. Hypertension is the largest contributor to deaths all over the world and serves as a modifiable risk factor for several health conditions, such as cardiovascular disease and renal dysfunction. With the treatment of hypertension, these health outcomes can be actively avoided. Lowering blood pressure through various lifestyle modifications or medications is an effective way of preventing hypertension and the morbidity and mortality associated with it. Some of the most effective treatment options include dihydropyridine and non-dihydropyridine CCBs. Although many studies have illustrated the effectiveness of these drugs, high blood pressure and hypertension continue to remain prevalent today. This may be due to a variety of reasons, some of which include low use of combination therapy and inadequate combination therapy optimization [2]. Both types of CCBs are effective as monotherapy or in combination with other drugs that work to reduce blood pressure. Dihydropyridine CCBs exert their desired effects by blocking smooth muscle L-type calcium channels, promoting peripheral vasodilation, and thereby lowering blood pressure. As a result of this vasodilation, CCBs may cause peripheral edema in patients. The excess accumulation of fluid serves as a contraindication for heart failure patients. Alternatively, non-dihydropyridine CCBs block cardiac calcium channels in addition to L-type calcium channels; therefore, they reduce heart rate and cardiac contractility in addition to lowering blood pressure. Other side effects involving both types of CCBs include constipation. Additionally, patients need to be monitored for drug interactions as they both inhibit the cytochrome P450 3A4 enzyme.

In addition to reducing blood pressure and preventing hypertension, CCBs also convey a cardioprotective effect. Short-acting CCBs prevent cardiac fibrosis and hypertrophy while also improving left ventricular function in heart failure patients after experiencing an MI [59]. Additionally, long-acting CCBs exert a decreased inotropic effect on the heart, decrease conduction through the AV node, and reduce myocardial oxygen consumption [39]. Therefore, these drugs may be useful in treating hypertension while also reducing the risk of future cardiovascular problems related to hypertension.

Of note, CCBs exert a more prominent response in hypertensive patients compared to normotensive patients, further demonstrating their use as antihypertensives. Although they can be used in monotherapy, CCBs are more effective when combined with other medications that affect the renin–angiotensin–aldosterone system (RAAS), such as ACE inhibitors or ARBS. Combination therapy with either of these drugs helps counteract the edema that may result from CCBs while also enhancing the antihypertensive properties of the drug. Alternatively, combination therapy may also include the use of a DHP and an NDHP, which confer certain benefits over the previously stated combination. The use of two CCBs does not exhibit the electrolyte abnormalities that occur with diuretic use, making them an ideal antihypertensive agent in patients with renal disease. However, further investigation is required to confirm the benefits of same-class combination therapy compared to the use of two different drug classes. Therefore, first-line combination therapy involving a CCB and an agent of another drug class remains the preferred treatment for refractory hypertension.

The scope of calcium channel blocker therapy for hypertension in individuals with other comorbidities is expanding. A retrospective cohort study conducted in 2021 revealed that dihydropyridine calcium channel blockers were linked to a reduced risk of Parkinson’s disease in patients newly diagnosed with hypertension (Tseng et al., 2021) [60]. In particular, hypertension diagnosed in middle age is correlated with a heightened risk of cerebrovascular disease and cognitive decline [60]. A longitudinal cohort study, conducted in Sweden, investigated the risk of death and ischemic stroke in patients with dementia and hypertension treated with CCBs [61]. They concluded that patients taking amlodipine, with Alzheimer’s dementia, Lewy body dementia, or Parkinson’s dementia, have a lower mortality risk than those taking other CCBs. Alzheimer’s dementia patients also taking amlodipine had a lower stroke risk [61]. CCBs are extensively utilized in various cardiovascular-related conditions, including arrhythmias, angina, and ischemic heart disease. For instance, nifedipine mitigates the effects of ischemic heart conditions by enhancing exercise-induced wall motion [62]. Additionally, they can be combined with beta-blockers to enhance cardiac output and are the primary treatment option for vasospastic angina [62]. CCBs are also categorized as class IV antiarrhythmics. By reducing conduction through the AV node, they disrupt re-entry circuits that can lead to supraventricular tachycardia, thus aiding in the management of arrhythmias [62]. Certain CCBs, such as amlodipine and lacidipine, have demonstrated efficacy in reducing atherosclerosis. Their mechanism involves reducing the oxidation of low-density lipids and preventing their deposition within arteries [62]. See Table 3.

## 5. Conclusions

While many studies demonstrate how CCBs should be included as first-line therapy for hypertensive patients, some research has demonstrated different effects of this class of medication amongst different ethnicities. For example, African Americans may have an improved response to CCB therapy alone when compared to combination therapy with diuretics. However, there is still a lack of significant data to conclude that CCB monotherapy is superior to combination therapy in African Americans. For many patients with only mild increases in systolic and diastolic blood pressure (e.g., stage 1 hypertension), the medical literature indicates that CCB monotherapy can be sufficient to control hypertension. In this regard, CCB monotherapy in those with stage 1 hypertension reduced renal and cardiovascular complications compared to other drug classes. Combination therapy with CCBs and angiotensin receptor blockers or angiotensin-converting enzyme inhibitors has been shown to be an effective dual therapy based on recent meta-analyses. Therefore, without sufficient data on the findings previously discussed, clinicians should not prescribe antihypertensive medications based on a patient’s ethnicity and should determine which medication or combination of medications is most appropriate based on individualized assessment.

## Figures and Tables

**Table 1:** Calcium channel blockers. This table compares the two classes of calcium channel blockers, dihydropyridines (DHPs) and non-dihydropyridines (Non-DHPs). This table elucidates the scope of variety within the two classes of calcium channel blockers.

Dihydropyridines	Non-Dihydropyridines
Amlodipine (Norvasc)	Diltazem (Cardizem and others)
Felodipine (Plendil)	Verapamil (Verelan)
Isradipine (DynaCirc, Prescal)	
Nicardipine (Cardene, Carden SR)	
Nifedipine (Procardia)	
Nisoldipine (Sular)	
Arandipine (Sapresta)	
Barnidipine (HypoCa)	
Benidipine (Coniel)	
Cilnidipine (Atelec, Cinalong)	
Clevidipine (Cleviprex)	
Efonidipine (Landel)	
Felodipine (Plendil)	
Lacidipine (Motens, Lacipil)	
Lercanidipine (Zanidip)	
Manidipine (Calslot, Madipine)	
Nilvadipine (Nivadil)	
Nimodipine (Nimotop)	

**Table 2:** Overview of side effects of different calcium channel blocker classes.

	Dihydropyridines	Non-Dihydropyridines
Examples	Amlodipine (Norvasc), Nifedipine (Procardia), Nicardipine (Cardene)	Diltiazem (Cardizem) and Verapamil (Verelan)
Vasodilation	Profound vasodilatory effect	Minimal vasodilatory effect
Chronotropy	No significant effect	Negative chronotropic effect
Inotropy	No significant effect	Negative inotropic effect
Other Side Effects	Peripheral edema, flushing, hypotension, gastroesophageal reflux, and gingival hyperplasia	Bradycardia, constipation, gastroesophageal reflux, and gingival hyperplasia

**Table 3:** Main findings from various studies cited in the literature.

Author, Year	Study Population	Results and Findings	Conclusions
Kalar HX, et al. 2021 [61]	Hypertensive dementia patents from the Swedish Dementia Registry (2008–2014)	For patients with hypertension and dementia, CCBs, specifically nifedipine, are associated with increased mortality risk. However, patients diagnosed with Alzheimer’s dementia, dementia with Lewy bodies, or Parkinson’s dementia taking amlodipine had a lower mortality risk [61].	Amlodipine is associated with reduced mortality risk in patients diagnosed with dementia (Alzheimer’s, Lewy body, or Parkinson’s dementia) [61].
Shah K, et al. 2022 [62]	Review of pharmacology targeting calcium channels in the heart	Calcium channel blockers aid in the treatment of various cardiovascular diseases but can have adverse effects including peripheral edema, constipation, and bradycardia [62].	Calcium channel blockers and their specificity for calcium channels in the vasculature and the heart make them excellent pharmacological treatments for angina, arrhythmias, hypertension, and atherosclerosis [62].
Tseng YF et al. 2021 [60]	Patient with Parkinson’s disease with newly diagnosed hypertension between 2001 and 2003	There were fewer cases of Parkinson’s disease in patients treated with dihydropyridine calcium channel blockers as compared to those who did not take DCCBs [60].	There was a significantly reduced risk of Parkinson’s disease in patients treated with dihydropyridine calcium channel blockers who were newly diagnosed with hypertension [60].
